# Massive Gastrointestinal Haemorrhage Unusual Presentation of Gastrointestinal Stromal Tumors of the Jejunum: Case Report and Literature Review

**DOI:** 10.7759/cureus.14266

**Published:** 2021-04-02

**Authors:** Abbas A Mohamed, Saleh M Al Zahrani, Sarah A Mohamed, Ahmad S Qureshi

**Affiliations:** 1 Department of General Surgery, National Guard Hospital, Al Madinah, SAU; 2 Department of General Surgery, University Hospital of Wales, Cardiff, GBR; 3 Department of Intensive Care Medicine, National Guard Hospital, Al Madinah, SAU

**Keywords:** gastrointestinal stromal tumors (gists), small bowel, massive bleeding

## Abstract

Although gastrointestinal stromal tumors (GISTs) are rare tumors, they are the most common tumors of mesenchymal origin of the gastrointestinal tract. GISTs present with nonspecific clinical manifestation and they are discovered incidentally during endoscopic or radiological investigations. Massive life-threatening bleeding that requires urgent surgery is rare. We present a case of small bowel GIST that presented with massive lower gastrointestinal bleeding that required urgent surgical intervention.

## Introduction

Gastrointestinal stromal tumors (GISTs) can occur anywhere throughout the gastrointestinal tract, and the small intestine is the second-most frequent site after the stomach [1]. GISTs of the jejunum are rare account for only 0.1%-3% of all gastrointestinal tumors [2]. A few cases (25%) present with melena, hematemesis, and anemia due to recurrent bleeding [3]. They rarely present with massive gastrointestinal hemorrhages requiring urgent intervention. We present a 58-year-old female who was presented with upper abdominal pain and black-colored stool for four days before her presentation to the emergency department. She also had symptoms and signs of anemia and massive lower GI hemorrhage. Colonoscopy and esophagogastroduodenoscopy failed to identify the source of the bleeding. Mesenteric CT angiogram showed a small soft tissue lesion in the proximal jejunum highly suggestive of GIST. She was successfully treated with laparoscopic assist resection.

## Case presentation

A 58-year-old female was admitted to the emergency department complaining of upper abdominal pain and the passage of the frequent large volume of black, tarry stool associated with palpitations, dizziness, and body weakness for four days. She denied a history of heartburn and chronic dyspepsia and she was not on aspirin or non-steroid anti-inflammatory drugs. She had a history of passage of black stool on and off for the last 6 months without being investigated. She was not diabetic or hypertensive and had no surgery before. On examination, she was pale, her pulse was 96 per minute and her blood pressure was 96/60 mmHg. A complete systemic examination revealed no pertinent findings apart from mild distension of the abdomen. Laboratory investigations showed hemoglobin of 6.2 G/del, hematocrit 29.6%, mean corpuscular volume (MCV) 64, mean corpuscular hemoglobin (MCH) 21.2, and mean corpuscular hemoglobin concentration (MCHC) 30%. The white blood cell count was 9.8 K/mm^3^ with 70% neutrophils and 18.4% lymphocytes and the platelet count was 357 x 10^9^/l. The coagulation screen showed an international normalized ratio (INR) of 0.96 seconds and prothrombin time (PTT) of 27.3 seconds. Other blood tests, including, urea, electrolytes, and liver function tests were within normal limits. 

**Figure 1:**
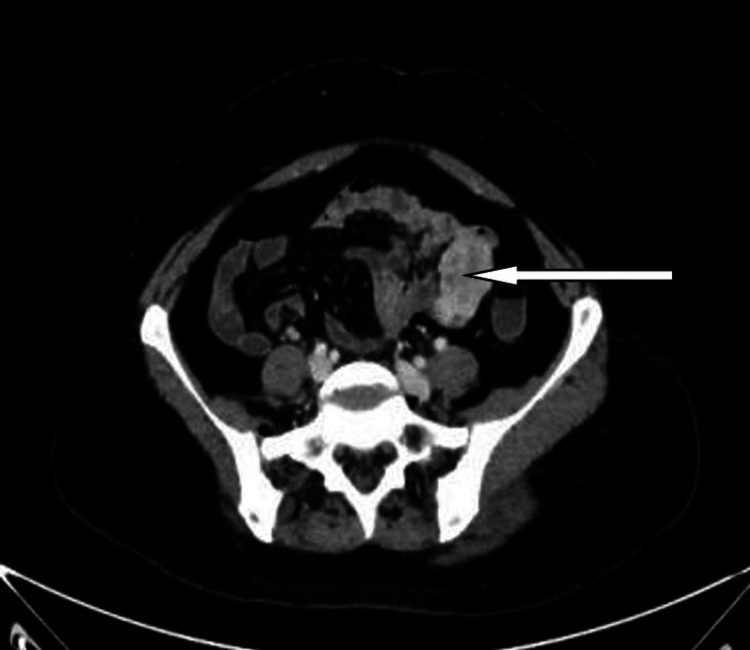
CT scan angiogram image showing a 4 x 3.7 x 3 cm heterogeneously enhancing soft tissue lesion arising from the proximal jejunum (the arrow).

**Figure 2:**
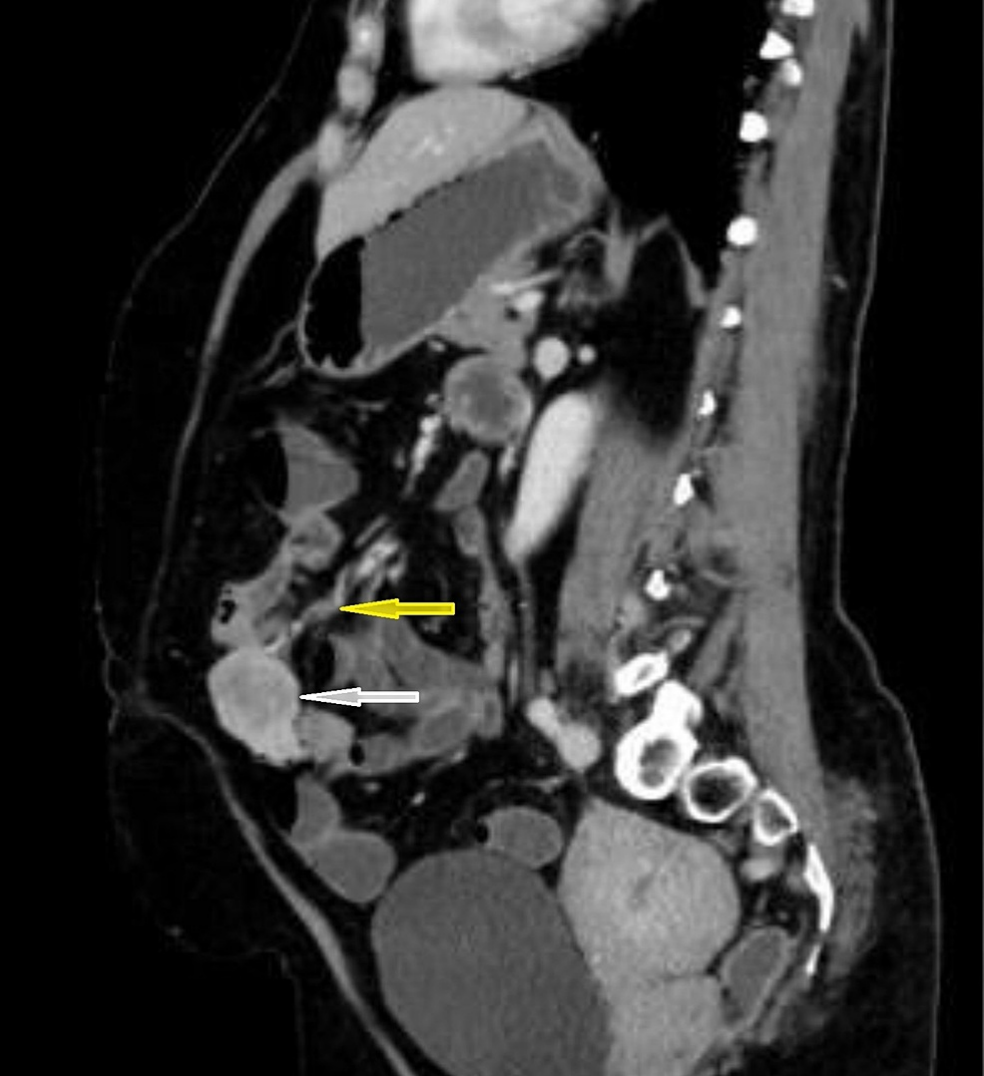
CT scan angiogram image showing the soft tissue lesion (the white arrow) with the feeding blood vessels from the central mesenteric blood vessels (the yellow arrow).

After initial fluid resuscitation and transfusing of three units of red blood cell the patient underwent urgent colonoscopy, and esophagogastroduodenoscopy that failed to identify the source of the bleed. An urgent mesenteric CT angiogram showed a 4 x 3.7 x 3 cm heterogeneously enhancing soft tissue lesion with feeding blood vessels from central mesenteric vessels at the level of the umbilicus. The lesion did not show evidence of active bleeding at the time of the study. There was no regional nodal involvement. No obvious hepatic focal lesions (Figures 1, 2).

**Figure 3:**
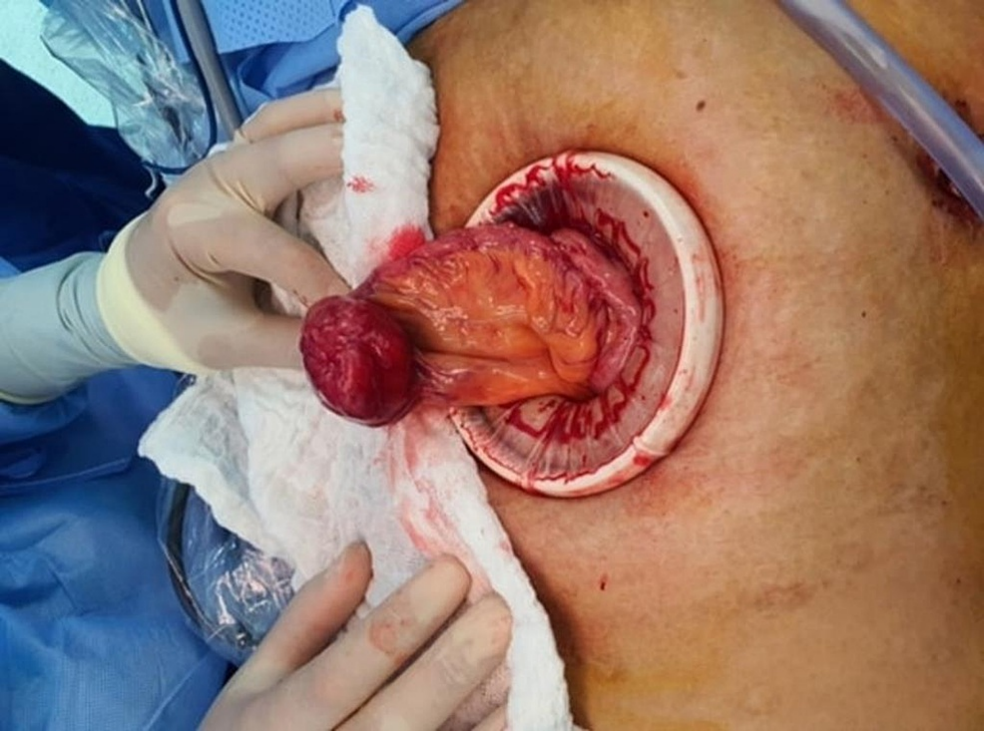
A photograph showing the tumor and the involved segment of the small bowel outside the abdomen.

After the patient was stabilized hemodynamically she was taken for urgent surgical exploration. The surgery was initially performed laparoscopically. The tumor was found arising from the proximal jejunum about 40 cm from Treitz's ligament. It was freely mobile with an intact serosal covering. There was no intraperitoneal hemorrhage. The tumor was grasped was exteriorized from the abdominal cavity through a 5 cm longitudinal incision immediately below the umbilicus (Figure 3).

**Figure 4:**
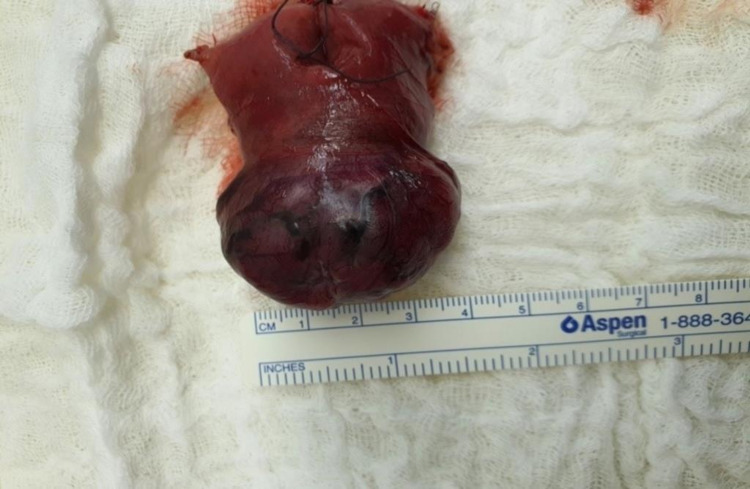
A photograph showing the resected tumor.

Resection was performed with a 5 cm clearance margin on each side of the tumor (Figure 4). The bowel anastomosis was performed side to side (Covidien-USA) Endo GIA™ 45mm stapler.

The post-operative period was uneventful. The patient was started on oral feeding on the 4th postoperative day and was discharged on the 6th post-operative day.

Macroscopically, the specimen consists of a segment of small bowel measuring 8.0 cm in length and 4.0 cm in width. Upon opening, there is a well-circumscribed polypoid pale pink tumor projecting into the bowel lumen and extending externally to the serosal surface. The tumor measures 4.0 x 3.0 x 2.0 cm in maximum dimensions and about 3.0 cm from each resection margin. The mucosa surface was lacerated containing clotted blood vessels, but the serosal surface appeared intact with no evidence of rupture. 

Microscopically the tumor was well-circumscribed exhibiting bland spindle cells with eosinophilic cytoplasm and a syncytial growth pattern. The tumor arises within the muscularis propria and replaced the full bowel wall thickness. The cell nuclei were elongated with inconspicuous nucleoli, with focal areas of nuclear palisading and interlacing fascicles of spindle cells with cigar-shaped nuclei. There was no significant nuclear pleomorphism or excessive mitotic activity. the tumor contains normal small intestinal mucosa with excessive ulceration.

The tumor cells showed strong positive immunostaining reactions with CD117 (C-kit), Bcl2, CD34, and SMA. Immunostaining reactions with Desmin, S100, and Melan-A were negative. The morphological appearances in conjunction with the immunohistochemical staining profile are those of a typical low-grade GIST.

## Discussion

GISTs are relatively rare tumors that originate from the interstitial cells of Cajal, which are located in and around the myenteric plexus and are thought to function as intestinal pacemaker cells [4]. Historically, GISTs were misclassified as leiomyomas or leiomyosarcomas. Subsequently, it was determined that GISTs have distinct immunophenotypical markers and ultrastructural features that differ from smooth muscle tumors [5]. Mazur and Clark were the first to report GIST as a separate entity from gastrointestinal smooth muscle tumors in 1983 [6]. GISTs are currently defined as gastrointestinal tract mesenchymal tumors containing spindle cells and showing CD 117 (c-kit protein) positivity in more than 95% of cases [7].

Although they can arise throughout the gut, the most common site is the stomach (52%), followed by the small intestine (25%) [8]. Jejuna GISTs are extremely rare, representing about 0.1%-3% of all gastrointestinal (GI) tumors [2].

Diagnosis of small GISTs of the small bowel is difficult because they remain silent or present with vague abdominal symptoms. However, with the increasing use of CT scans for investigating abdominal pathology, there is an increasing number of incidentally discovered GISTs. A few cases (25%) present with melena, hematemesis, and anemia due to recurrent bleeding [3]. Other presenting symptoms and signs include early satiety, abdominal pain, and a palpable mass. They can occasionally present as surgical emergencies such as bowel obstruction, perforation, or gastrointestinal haemorrhage [9].

Diagnosis of small bowel GISTs depends on a high index of suspicion. GISTs should be suspected in all patients presented with the triad of recurrent melena or positive hem occult stool test, unexplained anemia, and negative upper and lower GI endoscopy. CT scan provides the basis for diagnosis and staging in most patients. It is considered an investigation of choice as it provides a rapid and reproducible assessment of the size of the primary tumor, as well as its relationship to other structures. Metastatic disease is well be demonstrated at the outset [10].

Small primary GIST appears on non-enhanced CT scan as well delineated, low soft tissue density. On enhanced CT these tumors, when small, typically show homogeneous enhancement. The CT features are not diagnostic and GISTs can mimic more common tumors such as pancreatic, or esophageal cancer and small bowel GISTs can mimic bowel lymphoma [11]. Preoperative fine-needle aspiration is not advisable due to the risk of tumor rupture and intraperitoneal seeding [12].

Massive GI bleeding, which requires urgent surgical, endoscopic intervention, or arterial embolization is an extremely rare presentation of GISTs. GIST bleeding is intraluminal in most cases, however extraluminal bleeding and spontaneous hemoperitoneum were reported in few cases [13].

The mechanism of bleeding in GISTs is not yet well understood. The main causes of intraluminal bleeding of GISTs are related to mucosal and submucosal destruction by tumor growth and invasion of nutrient vessels l [14], while the cause of the extraluminal intraperitoneal bleeding is tumor rupture in most cases.

Both massive intraluminal and extraluminal bleeding tend to occur with relatively large GISTs (>2 cm), although a few cases of massive bleeding were reported with small tumors (<2 cm).

Our case is one of those rare presentations as our patient had small GIST of the jejunum presented with massive lower GI bleeding that required preoperative blood transfusion and urgent surgery.

Notani et al. [15] reported a case similar to our case of jejunal GIST presented with repeated melena that was diagnosed by CT scan and removed by laparoscopy-assisted surgery, while Shi et al. [9] reported a case of a 2.0 cm × 2.5 cm GIST of proximal jejunum presented with massive gastrointestinal bleeding that was successfully treated with endoscopic sclerotherapy.

Govindaraj et al. [16] reported another similar case of small jejunal GIST (2 cm) that presented with massive gastrointestinal hemorrhage that required urgent laparotomy. They suggested that incidentally detected small bowel GISTs need to be operated upon irrespective of their size at the time of detection, as these tumors can present as acute emergencies later.

Similarly, Khuri et al. [17] reported two cases of small bowel GIST presented with massive life-threatening hemorrhage that required urgent surgery.

The management of small (<2 cm) small-bowel GISTs is controversial. Most of the guidelines recommend that small asymptomatic GISTs can be managed conservatively. The American Gastroenterological Association recommends the removal of all GISTs with a diameter ≥of 3 cm [18]. The National Comprehensive Cancer Network recommends the removal of all GISTs with a size ≥of 2 cm [19], whereas the European Society for Medical Oncology recommends the removal of all GISTs >2 cm [20]. Unfortunately, these guidelines are primarily based on the risk of malignancy and there is no guideline predicting the risk of complications in small-bowel GISTs. Few authors [16] suggested the removal of these tumors even if they were incidentally discovered regardless of their size.

## Conclusions

Massive GI bleeding, which requires urgent intervention is a relatively rare presentation of GISTs. Most of the guidelines recommend that small asymptomatic GISTs can be managed conservatively, unfortunately, these guidelines are primarily based on the risk of malignancy and there is no guideline predicting the risk of complications such as hemorrhage. With increasing published reports of small GISTs presenting with life-threatening hemorrhage that requires urgent intervention, we recommend that all incidentally discovered GISTs should be removed regardless of their size because of their potentials for serious complications.
